# Protective effect of methanolic extract of Berberis integerrima Bunge. root on carbon tetrachloride-induced testicular injury in Wistar rats

**Published:** 2016-02

**Authors:** Fereshteh Rafiee, Vahid Nejati, Reza Heidari, Hossein Ashraf

**Affiliations:** *Department of Biology, Faculty of Science, Urmia University, Urmia, Iran.*

**Keywords:** *Berberis*, *Antioxidant*, *Carbon tetrachloride*, *Testis*, *Rats*

## Abstract

**Background:**

Tissue protective effect of compounds with antioxidant properties has been demonstrated. The alkaloids found in barberry root are considered as antioxidants.

**Objective:**

According to barberry protective effects in different tissues, in this study, the protective effect of Berberis integerrima Bge. root )MEBIR) was evaluated against CCl4-induced testicular damages in Wistar rats.

**Materials and Methods:**

40 mature male rats were randomly divided into 5 groups: 1: Normal control, 2: Sham: received CCl4 diluted in olive oil (50% v/v; 1ml/kg bw), intraperitoneally, twice a week for 4 weeks, 3 and 4: Sham rats treated with MEBIR (250 and 500 mg/kg bw) for 28 days, 5: Sham rats treated with silymarin (50 mg/kg bw) for 28 days. After 28 days, serum testosterone level, absolute testis weight, catalase activity, malondialdehyde level, and histological parameters were investigated.

**Results:**

In the treated rats with MEBIR (250 and 500 mg/kg bw) or silymarin (50 mg/kg bw), there was a significant increase in the absolute testis weight, testosterone level, seminiferous tubules diameter (p<0.001), thickness of the epithelium, tubule differentiation index) p<0.001), spermiogenesis index (p<0.001), the activity of catalase, and a significant decrease in interstitial tissue thickness (p<0.001) and malondialdehyde level in comparison with CCl4-treated group. The effect of the MEBIR at dose of 500 mg/kg bw is more than that of the standard drug, silymarin (50 mg/kg bw).

**Conclusion:**

From the results, it is suggested that the protective effects of MEBIR is possibly due to antioxidant effects of its bioactive compounds.

## Introduction

Male sexual derangement is caused by various agents such as alcoholism, drug abuse, smoking, some drugs and toxic chemicals (1). Carbon tetrachloride is one of the compounds that damage testis and other organs such as liver, kidney, brain, and lungs (2-4). Free radicals are produced from metabolism of carbon tetrachloride that binds to cell membrane causing oxidative damages (5). In the body, the construction of reactive oxygen species is an unavoidable result in aerobic organisms leads to the overproduction of unwanted reactions within the body and subsequent tissue damages (6). Antioxidants found in food and the body, even in small amounts, can protect the body against various types of oxidative damage induced by oxygen-free radicals (7). 

Berberis integerrima Bge. (Berberidaceae) is an important medicinal plant with yellow wood, obovate leaves, yellow flowers, and oblong red colored fruits. It grows in several regions of Iran, particularly in north and north east of the country (8). For different parts of barberry plant, various properties are listed and these properties have been demonstrated in many studies. Different types of alkaloids are obtained from the root, of which the most important is Berberine (9). Based on researches on Barberry root extract and Berberine (its main alkaloid), the following effects are considered: hypoglycemic, hypolipidemic, anti-inflammatory effects, and liver protection (9-12). Majd *et al* showed that Berberis integerrima Bge. has antioxidant and anticancer properties (13). Furthermore, Ashraf *et al* showed that Berberis integerrima Bge. has antihyperglycemic and antihyperlipidemic effects, preventive and therapeutic roles on the serum levels of glucose and lipids, preventive effects on liver injury, and improved renal dysfunction in diabetic rats (14-17).

Given that antioxidant and protective effects of alkaloids in various plants and on multiple tissues have been demonstrated and since barberry plant contains alkaloids, this study was performed for the first time to investigate the protective effect of Berberis integerrima Bge. root extract on carbon tetrachloride-induced toxicity in the testes of male rats.

## Materials and methods

**Animals**

Forty mature male rats (Wistar strain) weighing 220-250 gr were tested in this research study. The animals were housed in polypropylene cages with free access to standard laboratory rat food and faucet water and were maintained in standard conditions of temperature (22±2^o^C), light cycle (12 hr light/dark), and relative humidity (40-60%). In this study, all animal procedures were carried out using protocols approved by the local ethical committee of Urmia University of Medical Sciences.

**Plant collection and extraction**

Wild samples of Berberis integerrima Bge. roots were collected from the countryside of Bavanat city (Fars province, Iran). A specimen of plants was submitted at the herbarium of Faculty of Science, Urmia University (Iran), and it was identified by the Botany Department and impounded in the herbarium (No. 9059). Roots (after washing with cold water) were dried in the shade then were powdered by using mechanical grinding. 3 liters of 70% methanol were added to 1000 gr of powder of root to isolate polar and nonpolar compounds. After 72 hr, barberry extract was filtered through Whatman filter paper No.45 and Solvent (methanol 70%) was removed by using rotary vacuum evaporator. After removing the solvent from the extract, in order to eliminate residual water, the resulting mixture was in the oven for 5 hr at 100^o^C. The dried extract was stored at 4°C for further in vivo investigations. Specific concentrations of extracts were prepared using normal saline (18).

**Acute toxicity study**

Acute toxicity study of MEBIR was determined as per the OECD guideline No. 423 (Acute Toxic Class Method). It was observed that MEBIR was found safe up to dose of 2,500 mg/kg of body weight. The rats were observed continuously for 24 hr for behavioral, neurological and then at 24 and 72 hr for any lethality. Test extract was not lethal to the rats even at 2500 mg/kg dose. Hence, 1/10th (250 mg/kg bw) and 1/5th (500 mg/kg bw) of this dose were selected for further study (19).

**Experimental design**

Forty mature male Wistar rats were randomly divided into 5 groups (n=8) and were treated for four weeks as follows: Group I (Normal control) was treated with normal saline (10 ml/ kg bw). Group II (Sham) was injected intraperitoneally with CCl4 diluted in olive oil (50% v/v) at a dose of 1ml/kg bw twice a week for 4 weeks and received normal saline (10 ml/ kg bw) (20). Group III received CCl4 similar to group two and was treated with MEBIR (dissolved in normal saline) at doses of 250 mg/kg bw. Group IV received CCl4 similar to group two and was treated with MEBIR (dissolved in normal saline) at doses of 500 mg/kg bw. Group V (Standard group) received CCL4 similar to group two and standard drug silymarin at a dose of (50 mg /kg bw, dissolved in normal saline, intragastric (21). Animals received barberry extracts and silymarin daily by gavage for 4 weeks.

**Blood collection**

At the end of the study, animals were anesthetized with diethyl ether. Blood was collected from their heart by heparinized syringes and kept at 37^o^C for 30 min. The serum was separated by centrifugation (3000 rpm at 40^o^C for 15 min) and stored at -30°C to measure testosterone levels.

**Assessment of absolute testis weight and histological analysis**

The testes of rats were dissected out, cleared of fats and blotted free of blood and weighed with the help of a Sartorius digital balance. The left testes was fixed in 10% buffered neutral formalin and used for histological investigations. After fixation of the testes, the fixed tissues were embedded in paraffin, sectioned (6-8 μm) with a rotary microtome (GmbH, Germany) and stained with Hematoxylin and Eosin. Ultimately, tissue sections were evaluated under the light microscope (Dialux 20 EB) at×400 magnification. Furthermore, the right testes were washed in cold normal saline for analysis of tissue homogenates.

**Assessment of testosterone**

The testosterone level of serum was measured by using radioimmunoassay method and a special kit for rats (WHO/Sigma Asso-RFGC-78/549) (22).

**Assessment of catalase (CAT) activity and malondialdehyde (MDA) levels**

Homogenate of the testes were prepared (10%). It was centrifuged at 4°C for 10 minutes at 7000 rpm. Supernatant solutions were used to measure CAT activity and malondialdehyde (MDA) levels. The activity of Catalase (CAT) was estimated by the method of Gott (23). MDA as the final product of lipid peroxidation was measured in the testis tissue extracts by the method of Ester Bauer and Cheese man. MDA and thiobarbituric acid react at 90-100ºC and produces a pink-colored compound. The absorbance of the tissue samples were read at 532 nm (24).

**Determination of histological parameters**

The diameter of the seminiferous tubule was measured using scaled lens; small and large mean diameter of each tubule was calculated using the formula (25). The epithelial thickness in a micrometer at 400X magnification (from spermatogonia in basal membrane to the spermatids) was measured using the scaled lens. Also, the thickness of the interstitial tissue was measured using the scaled lens in a micrometer (26). To estimate tubule differentiation index (TDI) and spermiogenesis index (SPI), 100 cross-sections of seminiferous tubules were randomly analyzed in each rat. TDI is the percentage of seminiferous tubules containing at least four differentiated germ cells (27). 

For TDI calculation, seminiferous tubules containing more than three layers of differentiated germinal cells from spermatogonia type A were considered as positive TDI. SPI is the percentage of seminiferous tubules with normal spermiation (28). For SPI calculation, the seminiferous tubules containing sperm and tubules without sperm in cross-sections of seminiferous tubules were identified and counted. 

**Statistical analysis**

Data from experiments are reported as mean±SE. Data were analyzed by one-way ANOVA; a significant difference between groups was determined by the Tukey test. P<0.05 was considered as the criterion for significant differences.

## Results

**Absolute testis weight**

**Figure 1:**
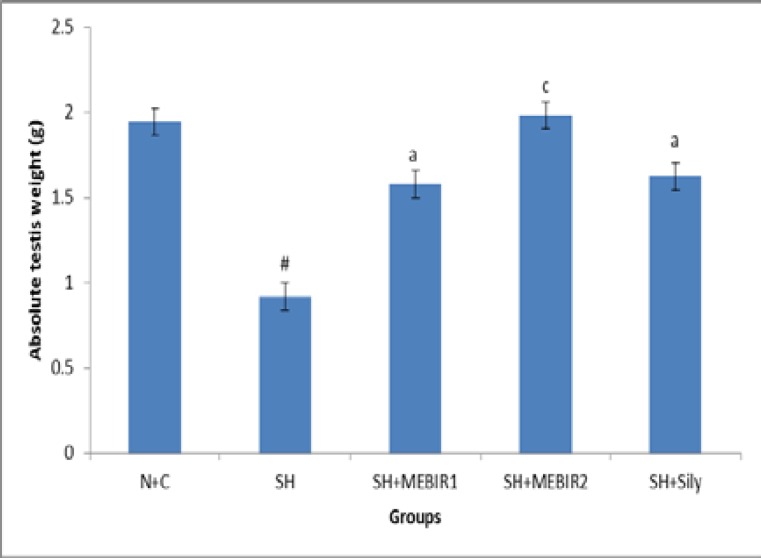
MEBIR 1 and 2: Methanolic extract of Berberis integer Rima root (250 and 500 respectively), N+C: Normal control, SH: Sham, SH+Sily: Sham rats treated with silymarin. Values are presented as mean±SEM; n=8 in each group. One-way ANOVA followed by Tukey test. #: p<0.001 sham rats were compared with normal control rats. a: p <0.05 and cp<0.001 sham rats treated with MEBIR 1 and 2 or silymarin were compared with untreated sham rats

As shown in figure 1, normal control animals were found to be stable in their testis weight. In sham rats, the testis weight was significantly decreased (p<0.001) in comparison to the normal rats. Rats treated with 250 and 500 mg/kg bw of MEBIR and silymarin (50 mg/kg bw) showed a significant increase in testes weight (71.73%, 115.21%, and 76.08% respectively) as compared with untreated sham rats.

**Serum testosterone level**

**Figure 2:**
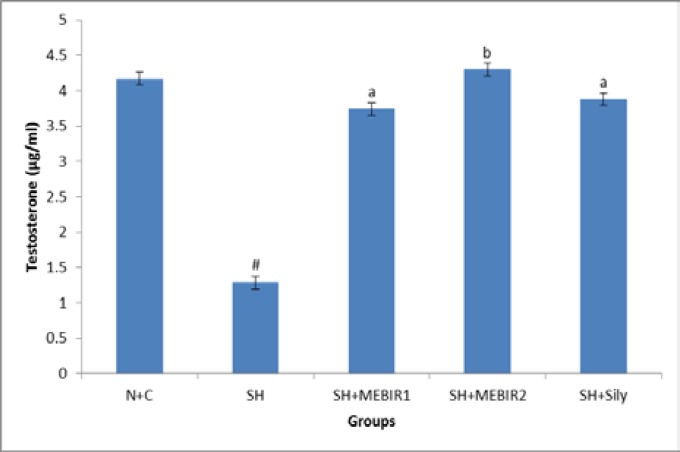
MEBIR 1 and 2: Methanolic extract of Berberis integerrima root (250 and 500 respectively), N+C: Normal control, SH: Sham, SH+Sily: Sham rats treated with silymarin. Values are presented as mean±S.E.M. n=8 in each group. One-way ANOVA followed by Tukey test. #: p<0.001 sham rats were compared with normal control rats. a: p<0.05 and b: p<0.01, sham rats treated with MEBIR 1 and 2 or silymarin were compared with untreated sham rats

The effect of MEBIR and silymarin on the serum testosterone level of normal and sham rats is shown in figure 2. In sham rats, the serum testosterone level was significantly decreased (p<0.001) in comparison to their normal levels. Administrating 250 and 500 mg/kg bw of MEBIR or silymarin (50 mg/kg bw) lead to significant increase in serum testosterone level in sham rats (189.92%, 233.33%, and 200.77% respectively) as compared with untreated sham rats.

**Histological parameters**

**Figure 3:**
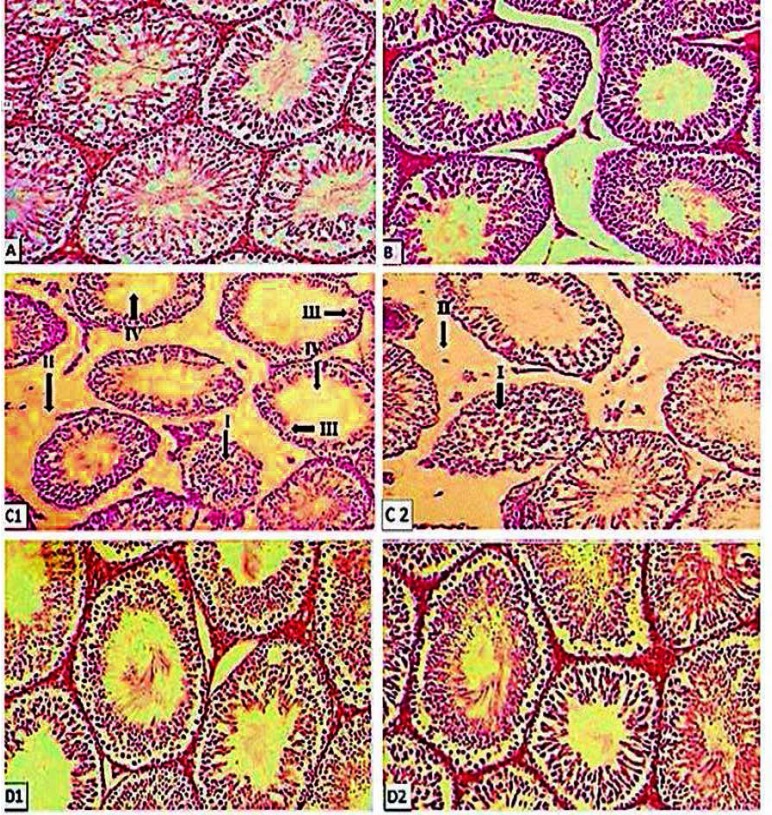
Histological sections of Wistar rats' testis in experiment groups.

Table I and figure 3 show the effects of MEBIR and silymarin on histological parameters of control and experimental groups. The seminiferous tubule diameter, epithelial thickness, tubule differentiation index (TDI), and spermiogenesis index (SPI) in sham in comparison to the normal control were decreased (p<0.001), but interstitial tissue thickness in sham in comparison to the normal control was increased (p<0.001). However, all these parameters, except interstitial tissue thickness, were increased significantly in sham rats treated with 250 and 500 mg/kg bw of MEBIR or silymarin (50 mg/kg bw) (19.79%, 21.02% and 17.62% for seminiferous tubule diameter, respectively), (61.98%, 99.17% and 78.51% for epithelial thickness, respectively), (229.34%, 269.98% and 191.31% for TDI, respectively), (and 294.38%, 346.25% and 245.70% for SPI, respectively) compared to the untreated sham rats, while the interstitial tissue thickness was decreased significantly (62.61%, 73.22% and 46.3%, respectively) compared to the untreated sham rats. Furthermore, the cell connections of seminiferous tubule were disturbed in sham rats while this change was improved in sham rats treated with 250-and 500-mg/kg bw of MEBIR or silymarin (50 mg/kg bw) (Figure 3).

**CAT activity and malondialdehyde (MDA) levels**

Table II shows the mean values of CAT activity and MDA level of both control and experimental groups after 4 weeks. In sham rats, the CAT activity significantly decreased (p<0.001), but MDA significantly increased (p<0.001) compared to the normal levels. However, the CAT activity significantly increased in sham rats treated with 250 and 500 mg/kg bw of MEBIR or silymarin (50 mg/kg bw) (106.83%, 134.61% and 98.29% for CAT respectively), compared to the untreated sham rats, while the MDA significantly decreased in sham rats treated with 250 and 500 mg/kg bw of MEBIR or silymarin (50 mg/kg bw) (35.46%, 42.38% and 26.40%, respectively) compared with untreated sham rats.

**Table I:** Effect of MEBIR and silymarine on histological parameters in sham rats

**Group**	**Treatment**	**Dose** **(mg/kg)**	**TDI** **(%)**	**SPI** **(%)**	**Epithelial thickness** **(µm)**	**Interstitial tissue thickness** **(µm)**	**Seminiferous tubules diameter (µm)**
1	N+C	10 ml/kg	90.68 ± 1.86	88.92 ± 2.64	46.60 ± 1.50	6.64 ± 0.58	259.40 ± 3.21
2	SH	10 ml/kg	23.72 ± 2.75#	18.16 ± 1.86#	24.20 ± 2.08#	24.50 ± 0.84#	211.20 ± 3.92#
3	SH+MEBIR	250	78.12 ± 2.78^c^	71.62 ± 3.54^c^	39.20 ± 2.22^a^	9.16 ± 0.58^c^	253.00 ± 5.66^c^
4	SH+MEBIR	500	87.76 ± 2.20^c^	81.04 ± 4.11^c^	48.20 ± 2.26^c^	6.78 ± 0.49^c^	255.60 ± 6.75^c^
5	SH+Sily	50	69.10 ± 5.26^c^	62.78 ± 4.60^c^	43.20 ± 4.25^b^	13.06 ± 1.61^c^	248.40 ± 7.07^c^

Values are presented as mean±SE. One-way ANOVA followed by Tukey test. (n=8)

SPI: Spermiogenesis index

N+C: Normal control

SH: Sham

Sily: Silymarin

MEBIR: Methanolic extract of Berberis integerrima root

TDI: Tubule differentiation index

#: p<0.001 sham rats were compared with normal control rats.

a : p<0.05,

b : p<0.01, and

c : p<0.001 sham rats treated with MEBIR or silymarin were compared with untreated sham rats.

**Table II:** Effect of MEBIR and silymarin on MDA and CAT in sham rats

**Group**	**Treatment**	**Dose(mg/kg)**	**MDA** **(nmol/mg tissue)**	**CAT** **(u/min)**
1	N+C	10 ml/kg	30.67 ± 2.36	5.77 ± 0.53
2	SH	10 ml/kg	58.93 ± 2.88#	2.34 ± 0.21#
3	SH+MEBIR	250	38.03 ± 3.57^b^	4.84 ± 0.55^a^
4	SH+MEBIR	500	33.95 ± 2.11^c^	5.49 ± 0.39^b^
5	SH+Sily	50	43.37 ± 3.34^a^	4.64 ± 0.22^a^

Values are presented as mean ± SE. MEBIR: Methanolic extract of Berberis integerrima root. (n=8)

One-way ANOVA followed by Tukey test;

CAT: catalase

MDA: malondialdehyde

N+C: normal control

SH: sham

Sily: silymarin

# p<0.001 sham rats were compared with normal control rats.

a p<0.05,

b p<0.01 and

c p<0.001 sham rats treated with MEBIR or silymarin were compared with sham rats.

## Discussion

In the present study, the activity of CAT enzyme and low level of MDA were inhibited by the effects of carbon tetrachloride on the testis, which is likely a direct toxic effect on the tissue and probably influence on gonadal reply to FSH and LH, that ultimately leading to reduced testosterone production (29). Furthermore, treated rats with MEBIR or with silymarin, showed a significant decrease in the level of MDA, and significant increase in the activity of CAT.

In the present study, CCl4-induced toxicity in rats was similar to the study reported by Khan and Ahmed (30). One of the common criteria for the diagnosis of tissue oxidative damage is the peroxidation of lipids in the form of aldehyde products such as MDA, which is one of the main reasons in the toxicity induced by carbon tetrachloride (31). Similar to the present study, Danladi *et al* showed that CCl4-induced toxicity increase in MDA level in the testes of the rats, and Curtis *et al *showed that, there was a reduction in the activity of CAT in sham rats (29, 32). In consonance with the present study, significant low MDA level was seen in groups treated with barberry extract, confirmed that the extracts are rich in antioxidants (9), and the use of barberry extracts prevented the decrease in CAT enzyme, which may be due to the elimination of free radicals by barberry extract. This leads to the preservation and conservation of this enzyme. This result is in agreement with other researches on antioxidant and elimination properties of barberry plant (13, 18).

It has been illustrated that, the reduction of CAT activity and increase in the MDA level, in toxicity induced by CCL4 in the testes of the rats, were improved by post administration of Launaea procumbens, and it is suggested that this protective effect is possibly due to antioxidant effects of its bioactive compounds (33). Moreover, present study showed that, the level of testosterone, absolute testis weight, SPI, TDI, seminiferous tubule diameter, and epithelial thickness decreased, and interstitial tissue thickness increased in sham rats.

We found that, treated rats with MEBIR or silymarin, showed a significant decrease in the interstitial tissue thickness, and a significant increase in the absolute testis weight, SPI, TDI, seminiferous tubules diameter and the level of testosterone, while the effect of the atrophy was not observed. Moreover, in the treated rats with MEBIR or silymarin, a close connection between the seminiferous tubules and different lines of sex cells in the seminiferous tubules, and the natural integrity in the testis were clearly observed. This effect is probably due to the antioxidant properties of barberry.

It has been shown that, cigarette smoke leads to reduction in seminiferous tubules diameter and seminiferous epithelial height. This reduction was improved by supplementation of honey, and it is suggested that this protective effect is due to antioxidant capacities of honey (34). On the other hand, Shalizar Jalali *et al*. showed that reduction in SPI, TDI, serum level of testosterone and absolute testis weight, in cyclophosphamide-induced toxicity, improved towards normality, by administration of Crataegus monogyna due to its antioxidant properties (35).

Moreover, Pashaeian *et al*. showed that after immobilization stress in rat, reducing interstitial tissue thickness in ginseng treatment group was observed (36). From the result of this study, and other findings about antioxidant properties of silymarin, it can be firmly claimed that the antioxidant activity of silymarin leads to improve the change observed in sham rats (37). Detrimental effects of carbon tetrachloride on the testes are well documented (30). Carbon tetrachloride is deposited in various tissues such as liver, brain, and testes because of the solubility in lipid and crossing of the cell membranes (38). Detoxification enzymes of the cytochrome P450 cause the production of secondary active and toxic metabolites, which can damage the testis and other body tissues (39).

Decomposition of carbon tetrachloride leads to the production of trichloromethyl and trichloromethyl peroxyl free radicals (40). It appears that the effect of free radicals causes the peroxidation of membrane lipid and unsaturated fatty acids as well as the reduction of antioxidant enzymes (38). It has been suggested that, one of the major causes of testicular damage is oxidative stress caused by production of free radicals, which causes lipid peroxidation, change in enzymatic activity and finally induces necrosis (41, 42).

CAT is an important enzyme that has been widely distributed in animal tissue, decomposes hydrogen peroxide and protects tissues against highly active hydroxyl radicals (43). Thus, the reduction of CAT activity may lead to some destructive effects of superoxide anion and hydrogen peroxide radicals. A significant decrease in testosterone leads to pathological changes in the testis tissue (44). The reduction of the seminiferous tubule diameter in sham rats indicates that the atrophy of these tubules leads to morphological and spermatogenesis disturbance in the testis. In fact, there is a positive relationship between the seminiferous tubules diameter and spermatogenesis activity (45).

The root and stem bark of barberry contain various alkaloids such as; Berberine, oxyacanthine, bermamine, palmatine, jatrorrhizine, columbamine, and berberubine which have antioxidant, anti-inflammatory, and therapeutic properties. Plant alkaloids are the most important antioxidant compounds. Considering the fact that barberry roots are rich in alkaloids and given that antioxidant properties of barberry root have been proven in many studies, we can say that the antioxidant activity of barberry root is probably related to its alkaloid compounds (9, 17, 46, 47)^9^).

## Conclusion

Based on our results, the barberry root could protect the testes of rats against CCl4-induced toxicity. Furthermore, the effect of barberry root extract (especially at doses of 500 mg/kg bw) was more than that of the standard drug, silymarin (50 mg/kg bw). Thus it could be suggested that, the extract of barberry root probably by its antioxidant properties protected the rat's testes against the toxic effects of carbon tetrachloride.

## Note

***This article extracted from M.Sc.*** ***thesis. (Fereshteh Rafiee)***
